# Dr. A. L. Hummel

**Published:** 1897-12

**Authors:** 


### Obituary.

Dr. A. L. Hummel, of Hummelstown, Pa., died at Denver, Col., October 24, 1897, in the thirty-ninth year of his age. He was born at Hummelstown, of sturdy Pennsylvania Dutch stock, received his preliminary education in his native state, and graduated in medicine at the University of Maryland, Baltimore, in 1884.

It was Dr. Hummel's first intention to engage in the practice of his profession, but he had a strong penchant for business, though he desired to cultivate it in relation to the guild of medicine. Medical journalism afforded an opportunity for him to exercise his talents in a manner satisfactory to himself, because it kept him in touch with doctors with whom he especially delighted to associate. He had previously been engaged in newspaper work, which adapted him all the more to his chosen field. He first undertook the publication and business management of a leading medical journal and after one or two changes in this branch of the work finally engaged in medical journal advertising, which latter he developed into a successful specialty. He was undoubtedly the best-informed man in this country on the subject of medical journals, and he was so far in the lead in this regard as to be in a class by himself.

He established the Hummel & Parmele medical advertising agency some years ago in Philadelphia, which was there conducted for a few years. Mr. Charles Roome Parmele, one of the partners, soon retired to engage in business in New York, though on the organisation of the A. L. Hummel advertising agency Mr. Parmele became vice-president. About two years ago the business was removed to New York, where it is now in successful operation.

Dr. Hummel began to develop signs of tuberculosis in 1894, since which time he has resided for the most part in Colorado, though he has made incursions into Mexico and Arizona at intervals, all with a view to regain health or prolong life. He was hopeful from the first, but latterly his intimate friends have seen that he was failing in strength and vigor and that the end was not far away. At last he died somewhat suddenly, though peacefully, and his remains were taken to his native place for interment. His faithful friend and former partner, Mr. Parmele, aided at the obsequies to assuage the grief of Dr. Hummel's widow, and has kept the business in such excellent condition that she will derive a substantial benefit therefrom.

Dr. Hummel was one of the most genial, generous-souled and companionable of men, bringing sunshine into every location where he went, and in his departure has left sadness in a wide circle of friends.

